# Prevalence and correlates of abscesses among a cohort of injection drug users

**DOI:** 10.1186/1477-7517-2-24

**Published:** 2005-11-10

**Authors:** Elisa Lloyd-Smith, Thomas Kerr, Robert S Hogg, Kathy Li, Julio SG Montaner, Evan Wood

**Affiliations:** 1 Department of Health Care and Epidemiology, Faculty of Medicine; University of British Columbia, 5804 Fairview Ave, Vancouver, Canada; 2 British Columbia Centre for Excellence in HIV/AIDS; St. Paul's Hospital, 608-1081 Burrard Street, Vancouver, Canada; 3 Department of Medicine; University of British Columbia, 3300 – 950 W. 10th Ave, Vancouver, Canada

## Abstract

Recent studies have indicated that injection-related infections such as abscesses and cellulitis account for the majority of emergency room visits and acute hospitalizations accrued by local injection drug users. The objective of this analysis was to examine the prevalence and correlates of developing an abscess among a cohort of injection drug users in Vancouver and to identify socio-demographic and drug use variables associated with abscesses at baseline. We examined abscesses among participants enrolled in a prospective cohort of injection drug users. Categorical variables were analyzed using the Pearson's chi-square test and continuous variables were analyzed using the Wilcoxon signed rank test. Among 1 585 baseline participants, 341 (21.5%) reported having an abscess in the last six months. In a logistic regression model that adjusted for all variables that were associated with having an abscess at p < 0.1 in univariate analyses, female gender [odds ratio (OR) = 1.7, [95%CI: 1.2 – 2.4]; p = 0.002), recent incarceration (OR = 1.7, [95%CI: 1.3 – 2.2]; p < 0.001), sex trade involvement (OR = 1.4 [95% CI: 1.0 – 2.0]; p = 0.03), frequent cocaine use (OR = 1.5 [95%CI: 1.2 – 2.0]; p = 0.002) and HIV serostatus (OR = 1.5, [95%CI: 1.2 – 2.0]; p = 0.003) were positively associated with having an abscess. Explanations for these associations require further study, and interventions are needed to address this highly prevalent concern.

## Findings

The Downtown Eastside of Vancouver, Canada is a community characterized by high rates of HIV among injection drug users (IDU), and is also the setting of one of North America's highest volume needle exchange program (NEP) [1]. Recent studies have indicated that injection-related infections, such as abscesses and cellulitis, account for the majority of emergency room visits and acute hospitalizations in local IDU [2,3]. Factors associated with the development of abscesses among IDU have not been well described in settings with widespread access to sterile injecting equipment and high rates of HIV infection. In particular, abscesses are not characterized in Vancouver. However, abscesses can lead to serious complications including but not limited to osteomyelitis [4], endocarditis [5-7], and septicemia [8,9]. An ongoing prospective cohort study of IDU in Vancouver allowed for an examination of the prevalence and factors associated having an abscess in this setting.

For these analyses, data was collected through the Vancouver Injection Drug Users Study (VIDUS), a prospective cohort that has been previously described in detail [1]. Data from participants who completed baseline questionnaires between May 1, 1996 and May 31, 2004 were evaluated for the present study. Participants were categorized on the basis of whether or not they reported having an abscess lasting for more than three days during the previous six months. Univariate and multivariable statistics were applied to determine factors associated with developing an abscess in the previous six months. Categorical variables were analyzed using the Pearson's chi-square test, and continuous variables were analyzed using the Wilcoxon signed rank test. Variables associated with having an abscess at p < 0.1 were considered in a subsequent logistic regression analysis.

Socio-demographic and drug-using characteristics considered in these analyses as potential risk factors included: age, gender, HIV status, unstable housing, residing in Vancouver's Downtown Eastside, incarceration in the previous six months, sex trade involvement, borrowing and lending of syringes, frequent heroin and cocaine injection, binge drug use, public drug injection, requiring help with injections, and methadone maintenance therapy use. Unstable housing was defined as living in a single room occupancy hotel, transitional living arrangements, or being homeless. Individuals who reported injecting cocaine or heroin once or more a day were defined as frequent heroin and cocaine injectors. Bingeing was defined as periods in which drugs were injected more often than usual. Variable definitions were consistent with previous analyses [1].

Overall, of the 1 585 baseline VIDUS participants, 341 (21.5%) reported having an abscess in the last six months. The factors associated with having an abscess at *p *< 0.1 in univariate analyses included: female gender (OR = 2.4, [95%CI: 1.8 – 3.0]; *p *< 0.001); unstable housing (OR = 1.3, [95%CI: 1.1 – 1.8]; *p *= 0.01); recent incarceration (OR = 1.7, [95%CI: 1.3 – 2.1]; *p *< 0.001); sex trade involvement (OR = 2.4, [95%CI: 1.9 – 3.1]; *p *< 0.001); frequent heroin use (OR = 1.4, [95%CI: 1.1 – 1.8]; *p *= 0.006); frequent cocaine use (OR = 1.9, [95%CI: 1.5 – 2.5]; *p *< 0.001); residing in Vancouver's Downtown Eastside (OR = 1.5, [95%CI: 1.1 – 1.9]; *p *= 0.003); and HIV serostatus (OR = 1.8, [95%CI: 1.4 – 2.3]; *p *< 0.001). Table 1 shows the baseline demographic characteristics of IDU stratified by having an abscess or not in the past six months for significant variables considered in the univariate analysis.

**Table 1:** Baseline demographic characteristics of IDU stratified by having an abscess in the past six months.

**Characteristic**	**No abscess past six months *n = 1 244***	**Abscess past six months *n = 341***	**Odds Ratio (95% CI)**	***p-*value**
**Gender**				
Male	848 (68.2)	162 (47.5)		
Female	396 (31.8)	179 (52.5)	2.4 (1.9 – 3.0)	< 0.001
**HIV status**				
Negative	919 (73.9)	209 (61.3)		
Positive	325 (26.1)	132 (38.7)	1.7 (1.4 – 2.3)	< 0.001
Unstable housing*				
No	492 (39.5)	109 (32.0)		
Yes	752 (60.5)	387 (68.0)	1.3 (1.1 – 1.8)	0.011
Recent incarceration*				
No	861 (69.2)	196 (57.5)		
Yes	383 (30.8)	145 (42.5)	1.7 (1.3 – 2.1)	<0.001
DTES residence*				
No	552 (44.4)	121 (35.5)		
Yes	692 (55.6)	220 (64.5)	1.5 (1.1 – 1.9)	0.003
Sex trade involved*				
No	942 (75.7)	191 (56.0)		
Yes	302 (24.3)	150 (44.0)	2.4 (1.9 – 3.1)	< 0.001
Heroin use*				
Less than daily	840 (67.5)	203 (59.5)		
Daily use	404 (32.5)	138 (40.5)	1.4 (1.1 – 1.8)	0.006
Cocaine use*				
Less than daily	858 (69.0)	182 (53.4)		
Daily use	386 (31.0)	159 (46.6)	1.9 (1.5 – 2.5)	< 0.001

Note: IDU = injection drug user, DTES = Downtown Eastside Residence. *Indicates behaviour during the six month period prior to the baseline interview.

As shown in Table 2, in a logistic regression model that adjusted for all variables that were associated with having an abscess at *p *< 0.1 in univariate analyses, female gender [odds ratio (OR) = 1.7, [95% CI: 1.2 – 2.4]; *p *= 0.002), recent incarceration (OR = 1.7, [95% CI: 1.3 – 2.2]; *p *< 0.001), sex trade involvement (OR = 1.4 [95% CI: 1.0 – 2.0]; *p *= 0.030), frequent cocaine use (OR = 1.5 [95% CI: 1.2 – 2.0]; *p *= 0.002) and HIV serostatus (OR = 1.5, [95% CI: 1.2 – 2.0]; *p *= 0.003) were independently and positively associated with having an abscess.

**Table 2:** Logistic regression of factors associated with having an abscess

**Characteristic**	**Odds Ratio**	**95% C.I.**	***p*-value**
**Gender**			
(Female vs Male)	1.7	(1.4 – 2.4)	0.002
**Frequent cocaine use**			
(Yes vs No)	1.5	(1.2 – 2.0)	0.002
**Recent incarceration**			
(Yes vs No)	1.7	(1.3 – 2.2)	<0.001
**Sex trade involvement**			
(Yes vs No)	1.5	(1.1 – 2.1)	0.030
**HIV serostatus**			
(Yes vs No)	1.5	(1.2 – 2.0)	0.003

Our results indicate female gender, recent incarceration, sex trade involvement, frequent cocaine use and HIV serostatus are positively associated with developing an abscess. These results are consistent with results from a study in Amsterdam where female gender and prostitution among women, as well as, frequent cocaine use were identified as independently and positively associated with skin abscesses [10]. In addition, the association between HIV-positive status and having an abscess has been noted elsewhere, and is understandable given that HIV-positive individuals may have a heightened susceptibility to bacterial infections [11,12]. Furthermore, high risk of infectious complications, such as endocarditis from abscesses [10], occur among HIV infected individuals [11].

Abscesses are a common consequence of injection drug use [13-15]. The present study demonstrates that widespread access to sterile syringes through high-volume needle exchanges and a medically supervised safer injection facility may not be sufficient to prevent high rates of abscesses among IDU in Vancouver. In addition, our findings demonstrate the need for educational and structural interventions to improve rates of sterile injecting [16,17].

Our study has limitations. First, although previous research has indicated that the VIDUS cohort is representative of Vancouver IDU [18], VIDUS is not a random sample. Second, the study relied on self-report, and therefore, the results could be susceptible to socially desirable reporting although we know of no reason why reporting abscesses would be subject to this concern. Third, the cross-sectional nature of this study precludes any conclusions regarding causal relationships between the variables studied and the outcome of interest. Further prospective study is needed to assess predictors of abscess in this setting.

In summary, 21.5% of IDU participating in this study reported having an abscess in the previous six months. Results from this study indicate female gender, recent incarceration, sex trade involvement, frequent cocaine use and HIV serostatus are independently and positively associated with developing an abscess in injection drug users. Given the potential health complications arising from bacterial infections our findings highlight the need for the expansion of programs to promote safer injection practices.

## Competing interests

The author(s) declare that they have no competing interests.

## Authors' contributions

Elisa Lloyd-Smith, Thomas Kerr and Evan Wood designed the study. Kathy Li conducted the statistical analysis. Elisa Lloyd-Smith drafted the manuscript and incorporated all suggestions. All coauthors made significant contributions to the conception and design of the analyses, interpretation of the data and drafting of the manuscript, and they all approved the version to be published.

## References

[B1] Wood E, Tyndall MW, Spittal PM, Li K, Hogg RS, Montaner JS, O'Shaughnessy MV, Schechter MT (2002). Factors associated with persistent high-risk syringe sharing in the presence of an established needle exchange programme. Aids.

[B2] Kerr T, Wood E, Grafstein E, Ishida T, Shannon K, Lai C, Montaner J, Tyndall MW (2004). High rates of primary care and emergency department use among injection drug users in Vancouver. J Public Health (Oxf).

[B3] Palepu A, Tyndall MW, Leon H, Muller J, O'Shaughnessy MV, Schechter MT, Anis AH (2001). Hospital utilization and costs in a cohort of injection drug users. Cmaj.

[B4] Roszler MH, McCarroll KA, Donovan KR, Rashid T, Kling GA (1989). The groin hit: complications of intravenous drug abuse. Radiographics.

[B5] DeWitt DE, Paauw DS (1996). Endocarditis in injection drug users. Am Fam Physician.

[B6] DiNubile MJ (1994). Short-course antibiotic therapy for right-sided endocarditis caused by Staphylococcus aureus in injection drug users. Ann Intern Med.

[B7] Miro JM, Moreno A, Mestres CA (2003). Infective Endocarditis in Intravenous Drug Abusers. Curr Infect Dis Rep.

[B8] Hankins C, Palmer D, Singh R (2000). Unintended subcutaneous and intramuscular injection by drug users. Cmaj.

[B9] Williamson N, Archibald C, Van Vliet JS (2001). Unexplained deaths among injection drug users: a case of probable Clostridium myonecrosis. Cmaj.

[B10] Spijkerman IJ, van Ameijden EJ, Mientjes GH, Coutinho RA, van den Hoek A (1996). Human immunodeficiency virus infection and other risk factors for skin abscesses and endocarditis among injection drug users. J Clin Epidemiol.

[B11] Selwyn PA, Alcabes P, Hartel D, Buono D, Schoenbaum EE, Klein RS, Davenny K, Friedland GH (1992). Clinical manifestations and predictors of disease progression in drug users with human immunodeficiency virus infection. N Engl J Med.

[B12] Flanigan TP, Hogan JW, Smith D, Schoenbaum E, Vlahov D, Schuman P, Mayer K (1999). Self-reported bacterial infections among women with or at risk for human immunodeficiency virus infection. Clin Infect Dis.

[B13] Brown PD, Ebright JR (2002). Skin and Soft Tissue Infections in Injection Drug Users. Curr Infect Dis Rep.

[B14] Murphy EL, DeVita D, Liu H, Vittinghoff E, Leung P, Ciccarone DH, Edlin BR (2001). Risk factors for skin and soft-tissue abscesses among injection drug users: a case-control study. Clin Infect Dis.

[B15] Binswanger IA, Kral AH, Bluthenthal RN, Rybold DJ, Edlin BR (2000). High prevalence of abscesses and cellulitis among community-recruited injection drug users in San Francisco. Clin Infect Dis.

[B16] Ross MW, Wodak A, Stowe A, Gold J (1994). Explanations for sharing injection equipment in injecting drug users and barriers to safer drug use. Addiction.

[B17] Wood E, Kerr T, Montaner JS, Strathdee SA, Wodak A, Hankins CA, Schechter MT, Tyndall MW (2004). Rationale for evaluating North America's first medically supervised safer injecting facility. Lancet Infect Dis.

[B18] Tyndall MW, Craib KJ, Currie S, Li K, O'Shaughnessy MV, Schechter MT (2001). Impact of HIV infection on mortality in a cohort of injection drug users. J Acquir Immune Defic Syndr.

